# Gut microbiota dynamics and metabolic pathways associated with bleomycin-induced pulmonary fibrosis progression

**DOI:** 10.7717/peerj.21693

**Published:** 2026-09-08

**Authors:** Jinpeng Cong, Wenjuan Xu, Yuzhu Zhang, Xiaoqian Ding, Shichao Cui, Xiaosa Chi, Xiaohui Yang

**Affiliations:** The Affiliated Hospital of Qingdao University, Qingdao, Shandong, China

**Keywords:** Pulmonary fibrosis, Gut microbiota, Metagenomics, Dysbiosis, Metabolic pathways, Gut-lung axis, Mouse model

## Abstract

**Background:**

Pulmonary fibrosis (PF) is a progressive respiratory disease characterized by epithelial injury, aberrant repair and excessive extracellular matrix deposition. Although the gut–lung axis is increasingly implicated in respiratory disorders, stage-resolved characterization of gut microbiota taxonomic and functional potential during PF development is limited.

**Methods:**

We established a bleomycin-induced murine PF model and performed cross-sectional shotgun metagenomic sequencing of fecal samples from separate cohorts at three defined stages: baseline (control), day 7 (early fibrosis; M7), and day 14 (established fibrosis; M14). Microbial taxonomy, alpha/beta diversity, and predicted functional capacity were inferred using Kyoto Encyclopedia of Genes and Genomes (KEGG) and Carbohydrate-Active enZymes (CAZy) annotations; associations were assessed using Procrustes and Spearman correlation analyses.

**Results:**

Histopathology and immunohistochemistry confirmed progressive fibrogenesis with increased TGF-*β*1 and *α*-SMA expression. Compared with baseline, bleomycin-treated groups exhibited stage-specific shifts in gut microbial composition, including depletion of mucin-associated taxa (*e.g.*, *Prevotella, Akkermansia muciniphila*) and expansion of *Muribaculaceae*- and *Clostridiaceae*-affiliated taxa. Alpha and beta diversity metrics differed across groups. KEGG/CAZy-based annotations revealed predicted, stage-dependent changes in microbial metabolic potential, including early reductions in pathways related to amino acid and glycan metabolism (M7) and later increases in predicted starch/sucrose catabolism, phosphotransferase system (PTS) representation, and secondary bile acid biosynthesis (M14). Correlation analyses linked compositional shifts to these predicted functional changes.

**Conclusion:**

In a stage-resolved, cross-sectional study, bleomycin-associated pulmonary fibrosis was accompanied by compositional and predicted functional alterations in the gut microbiota. These data identify candidate taxa and predicted pathways for follow-up mechanistic testing, but functional (metabolomic) and causality experiments are required to confirm whether and how microbial changes contribute to PF pathogenesis.

## Introduction

Pulmonary fibrosis (PF) is a chronic, progressive, and severe disease characterized by progressive collapse of lung tissue and impaired lung function (Koudstaal et al., 2023; Noble, Barkauskas & Jiang, 2012). Pulmonary fibrosis is driven by a complex, multifactorial pathogenic cascade. Current paradigms emphasize repeated or persistent epithelial injury followed by aberrant repair responses, persistent fibroblast and myofibroblast activation, and excessive extracellular matrix deposition. Immune dysregulation, chronic inflammation and oxidative stress contribute to this process, while emerging evidence also implicates metabolic dysfunction, barrier perturbation and microbiota-derived signals as additional modifiers of fibrogenesis (Kinoshita & Goto, 2019; Otoupalova et al., 2020; Wakwaya & Brown, 2019). Currently, despite significant advances in understanding the mechanisms of PF, effective therapeutic interventions remain scarce, necessitating further exploration of novel treatment approaches.

The gut microbiota, a community of bacteria and other species residing in the human intestine, plays a crucial role in maintaining host homeostasis and immune balance (Lu et al., 2022; Pickard et al., 2017; Wastyk et al., 2021). Recent studies have highlighted the significant contribution of gut microbiota dysbiosis to the development and progression of various chronic diseases, including respiratory disorders such as asthma, chronic obstructive pulmonary disease (COPD), and lung cancer (Hou et al., 2022; Sun et al., 2024a). In COPD patients, long-term antibiotic use or smoking leads to gut microbiota imbalance, triggering bacterial translocation to the lungs and exacerbating local infection and inflammation (De Nuccio, Piscitelli & Toraldo, 2022). The severity of asthma is associated with *Akkermansia* of the phylum *Verrucomicrobia*; supplementation with *Akkermansia* reduces airway inflammation (Michalovich et al., 2019). These findings underscore the profound impact of gut microbiota imbalance on respiratory health and disease progression.

PF, a common and serious respiratory disease, can lead to changes in the normal tissue structure of the lungs, decreased lung function, resulting in bodily hypoxia, and in severe cases, may lead to death. It has received considerable attention due to its profound impact on respiratory health and high mortality rate worldwide (Moon et al., 2021; Salisbury & Wijsenbeek, 2021). Existing research has implicated gut microbiota in the regulation of immune responses and mucosal inflammation in PF (Chen, Sun & Xu, 2024; Moss, Ryter & Rosas, 2022). Animal studies have shown that gut microbiota dysbiosis associated with early-life antibiotic exposure in mice can promote the development of skin and lung fibrosis symptoms in adulthood. In cystic fibrosis (CF) patients, the abundance of *Staphylococcus*, *Streptococcus*, and *Veillonella* in the gut microbiota is significantly increased, while the abundance of *Bacteroides*, *Bifidobacterium adolescentis*, and *Faecalibacterium prausnitzii* is significantly decreased (Enaud et al., 2019). Furthermore, the gut microbiota abundance is closely related to the patient’s lung function, disease exacerbation, and disease severity. Streptomycin treatment can significantly reduce the abundance of intestinal Lactobacillus in CF model mice and inhibit airway hyperreactivity in the lungs by suppressing the growth of this bacterium and promoting the proliferation of T cells such as Th17 cells (Bazett, Bergeron & Haston, 2016). However, little is currently known about the dynamic changes in gut microbiota composition and their potential specific mechanisms during the progression of PF. In the face of this knowledge gap, the urgency of further research on the role of gut microbiota in PF is highlighted.

Although prior studies have reported associations between gut microbiota alterations and pulmonary fibrosis in clinical and experimental settings (Gong, Song & Su, 2021; Li et al., 2024; Liu et al., 2024), detailed, stage-resolved shotgun metagenomic profiling across defined early and established fibrosis stages in a single model remains limited. In this study, we therefore aimed to provide high-resolution, stage-specific characterization of gut microbial taxonomic composition and predicted functional gene content in a bleomycin model, to identify candidate taxa and predicted metabolic pathways that co-vary with the fibrotic phenotype and that warrant mechanistic follow-up.

## Methods

### Animal experimental design

Specific-pathogen-free (SPF) C57BL/6J male mice (approximately 8 weeks old) were obtained from Beijing Vital River Laboratory Animal Technology Co., Ltd. All animal experiments were conducted in strict accordance with the Guide for the Care and Use of Laboratory Animals (National Research Council Committee for the Update of the Guide for the C, and Use of Laboratory A, 2011) and approved by the Ethics Committee of the Affiliated Hospital of Qingdao University (Ethics No. QYFYW2LL26275), following the ARRIVE guidelines. Mice were acclimated for 7 days prior to experimentation under controlled environmental conditions: temperature 22–25 °C, relative humidity 46–65%, and a 12-h light/dark cycle (lights on at 08:00, off at 20:00). Each mouse was housed individually in a ventilated cage (300 mm × 180 mm × 140 mm) to minimize inter-animal stress and cross-contamination. Environmental enrichment included a cardboard tunnel (100 mm long × 50 mm diameter) for burrowing, a crinkled-paper nestlet (5 cm × 5 cm) for nesting, and hardwood chew sticks to maintain dental health. Mice had ad libitum access to irradiated SPF rodent chow and autoclaved reverse osmosis water. Corncob bedding (5–8 mm particle size) was changed twice weekly, and temperature, humidity, and ammonia levels were recorded daily.

Mice were randomly assigned to three groups (*n* = 10 per group): Control (intratracheal saline), M7 (bleomycin, 2.0 mg/kg, analyzed at day 7), and M14 (bleomycin, 2.0 mg/kg, analyzed at day 14). Importantly, this study used a cross-sectional time-course design in which separate cohorts of animals were euthanized at the three time points, rather than repeated sampling of the same individuals over time. Because saline-treated control animals were sampled only at baseline (day 0), time-matched vehicle controls at days 7 and 14 are not included in the current design (see Discussion for limitations). Although no formal *a priori* power analysis was conducted, the group size of *n* = 10 per treatment aligns with standard practices in animal studies and is generally considered sufficient to detect biologically meaningful differences under conditions of acceptable variability. The group allocation was known only to the investigator responsible for randomization. All personnel involved in animal handling, outcome assessment, and data analysis were kept blind to the group assignments. Primary outcomes included: (1) severity of pulmonary fibrosis assessed by histopathological scoring of Masson’s trichrome-stained sections; (2) expression levels of fibrosis-related proteins determined by immunohistochemical detection of transforming growth factor-*β*1 (TGF-*β*1) and *α*-smooth muscle actin (*α*-SMA) in lung tissue; and (3) structural and functional changes in the gut microbiota evaluated by metagenomic sequencing of fecal DNA.

All invasive procedures were performed after intraperitoneal injection of 1% sodium pentobarbital (10 μL/g), selected for its rapid onset (3–5 min) and minimal respiratory depression, as confirmed in pilot studies. Fecal samples were collected non-invasively from the cage floor 24 h prior to scheduled euthanasia (day 6 for M7, day 13 for M14) to reduce stress-related microbial shifts; this timing was chosen to avoid terminal physiological stress and to obtain more representative gut microbiota profiles. On days 0 (Control), 7 (M7), and 14 (M14), mice were fasted for 12 h before sample collection. Under deep anesthesia, both lungs were rapidly excised. One lobe was immediately fixed in 4% paraformaldehyde for histopathological and immunohistochemical examination, while the contralateral lobe was snap-frozen in liquid nitrogen for metagenomic analysis.

Euthanasia was performed *via* intraperitoneal administration of pentobarbital sodium (150–200 mg/kg) once mice exhibited loss of corneal reflex, absence of spontaneous respiration, and fixed, dilated pupils. Death was confirmed by auscultation of ceased heartbeat and onset of rigor mortis within 15 min. No animals required premature euthanasia; all mice survived to their designated endpoints without showing severe distress (*e.g.*, dyspnea, marked lethargy, or >20% body weight loss) during daily welfare checks. At the end of the study, surviving mice were euthanized using the same pentobarbital overdose protocol, and carcasses were incinerated at the institution’s licensed biological waste disposal facility in compliance with local biosafety regulations.

### Pathological and immunohistochemical analysis

Paraffin-embedded tissue blocks were sectioned into adjacent serial slices for immunohistochemical staining of TGF-*β*1 and *α*-SMA, respectively. Stained slides were fully digitized using a PANORAMIC DESK/MIDI/250/1000 scanner (3DHISTECH, Budapest, Hungary), and representative fields were captured at high resolution. Quantitative analysis of immunopositive signal intensity was performed using the scanner’s integrated image analysis software, enabling objective comparison of protein expression among experimental groups.

### Metagenomic sequencing and analysis

Total metagenomic DNA was extracted from fecal samples using the E.Z.N.A. Soil DNA Kit (Omega Bio-Tek, Norcross, GA, USA), following the manufacturer’s protocol as previously described (Li et al., 2022). DNA integrity and concentration were verified by 1% agarose gel electrophoresis and NanoDrop 2000 spectrophotometer (Thermo Fisher Scientific, Waltham, MA, USA), respectively. Paired-end sequencing (400 bp fragments) was subsequently performed on the NovaSeq X platform (Illumina Inc., San Diego, CA, USA) at Majorbio Bio-Pharm Technology Co., Ltd. (Shanghai, China).

Low-quality reads were removed using fastp (v0.23.2). Relative abundances of microbial taxa were estimated based on the total number of classified reads per sample. Taxonomic classification of metagenomic reads was performed using Kraken2 (v2.1.1) against a comprehensive reference database, following procedures previously described (Buytaers et al., 2021). Relative species abundances were subsequently re-estimated using Bracken (v2.6.2), providing the species-level abundance distributions used for downstream diversity analyses. Representative sequences from the non-redundant gene catalog were aligned to the NCBI non-redundant database for taxonomic annotation and Cluster of Orthologous Groups (COG) analysis of proteins. Functional annotations using Kyoto Encyclopedia of Genes and Genomes (KEGG) and Carbohydrate-Active enZymes (CAZy) were used to infer the presence and relative abundance of genes associated with metabolic pathways. It should be noted that these annotations infer genomic potential (*i.e.,* predicted functional capacity) from gene content and do not constitute direct measurements of metabolic activity or metabolite concentrations. Accordingly, downstream interpretation of pathway results treats them as predicted metabolic potential requiring independent metabolomic validation.

### Statistical analysis

Alpha diversity was assessed using the Shannon, Simpson, and Chao1 indices to characterize within-sample microbial richness and evenness, as previously described (Mao et al., 2025). Between-sample (beta) diversity was visualized by principal coordinate analysis (PCoA). Differences in community composition across groups were statistically evaluated using PERMANOVA *via* the adonis function in the R package *vegan*. Differentially enriched microbial biomarkers were identified using Linear Discriminant Analysis Effect Size (LEfSe), implemented through the lefser function in the R package *lefser*.

Continuous variables were expressed as median (interquartile range, IQR), whereas categorical variables were presented as number (percentage). Between-group comparisons were performed using the chi-squared test or Fisher’s exact test for categorical variables, and the Mann–Whitney *U* test or Kruskal–Wallis test for continuous variables, as appropriate. The R software’s ggplot2 tool was used for visualizing data.

## Results

### The establishment of pulmonary fibrosis mouse models induced by bleomycin

**Figure 1:**
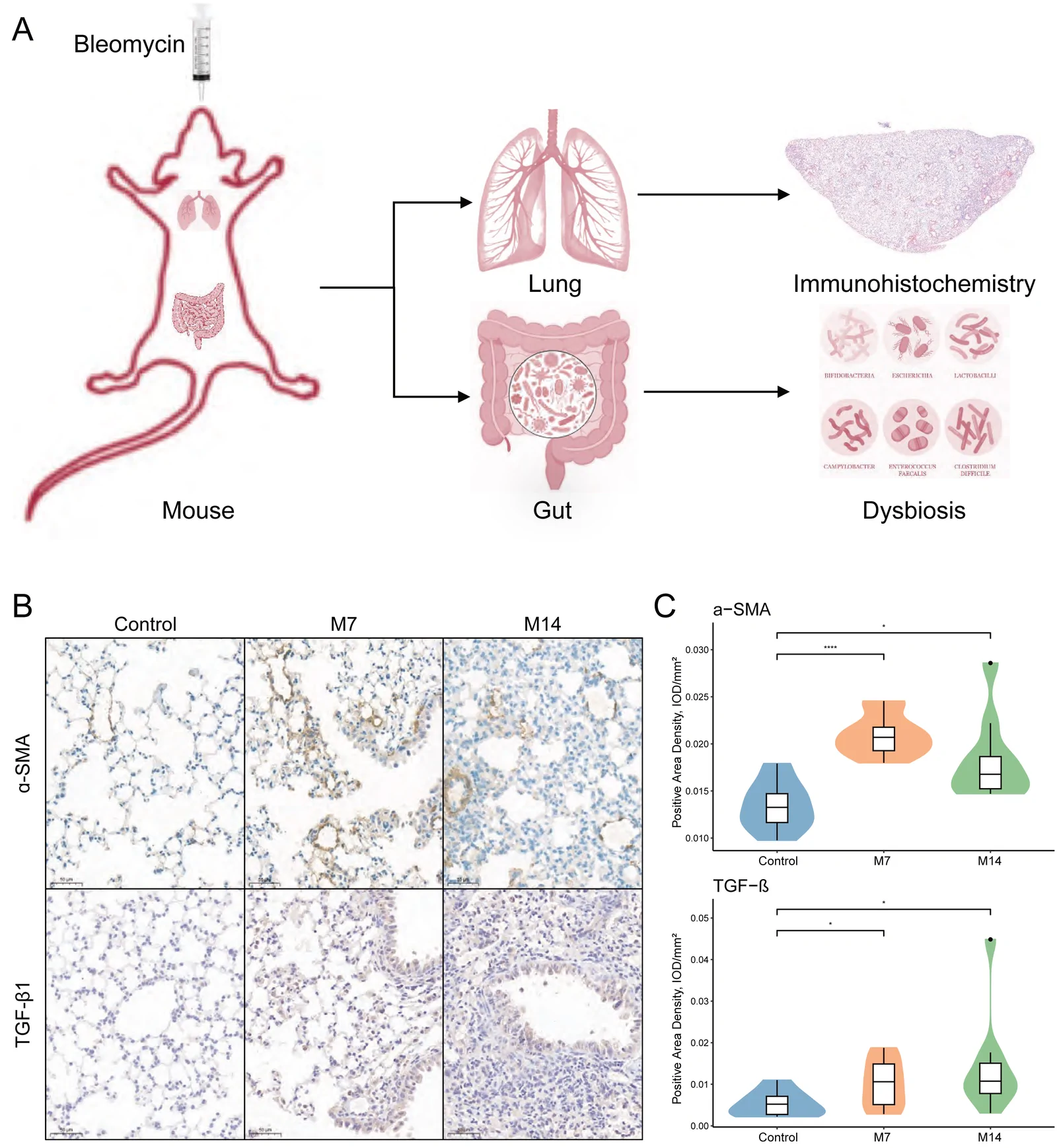
Bleomycin-induced pulmonary fibrosis mouse models. (A) Experimental design and timeline. Anesthetized mice (*n* = 10 per group) received intratracheal instillation of either saline (Control, 0.1 mL/10 g body weight) or bleomycin (2.0 mg/kg in saline; M7 and M14 groups) at day 0. Fecal samples were collected non-invasively 24 h prior to euthanasia (day 6 for M7, day 13 for M14, to minimize stress-related microbial shifts). Lung tissue and serum were collected at days 7 (M7, early fibrosis stage) and 14 (M14, established fibrosis stage) for histopathological, immunohistochemical, and metagenomic analysis. (B) Representative immunohistochemical staining of lung tissue. TGF-*β*1 and *α*-SMA staining of lung sections from Control, M7 (day 7), and M14 (day 14) groups. Lungs were perfusion-fixed with 10% buffered formalin and sectioned at five µm thickness. Original magnification, 20×. (C) Quantification of TGF-*β*1 and *α*-SMA expression in lung tissue by immunohistochemistry. Each point represents an individual animal (*n* = 10 per group). Bars indicate mean ± SEM. Signal intensity was quantified using integrated image analysis software on a PANORAMIC DESK/MIDI/250/1000 scanner. Statistical analysis: Kruskal–Wallis test with Dunn’s post hoc correction. *p < 0.05, ****p < 0.0001 *versus* Control.

To comprehensively investigate the impact of gut microbiota changes on lung fibrosis, this study employed the injection of bleomycin (2.0 mg/kg) into the airway of the mouse (Fig. 1A), aiming to simulate or observe the effects of gut microbiota on lung tissue. Compared to the control group, the injection of bleomycin did not significantly affect the body weight and food intake of the mouse (*P* > 0.05). Based on the clinical stages of lung fibrosis, at days 7 (M7) and 14 (M14), lung tissue and feces were collected from 10 mice. Immunohistochemistry results revealed that TGF-*β*1 (a key cytokine) in the lung tissue significantly increased with advancing fibrosis, and the expression of *α*-SMA (a fibrosis-related marker) also significantly increased (*P* < 0.05, Figs. 1B and 1C).

### Significant structural differences in gut microbiota of mouse fibrosis model

**Figure 2:**
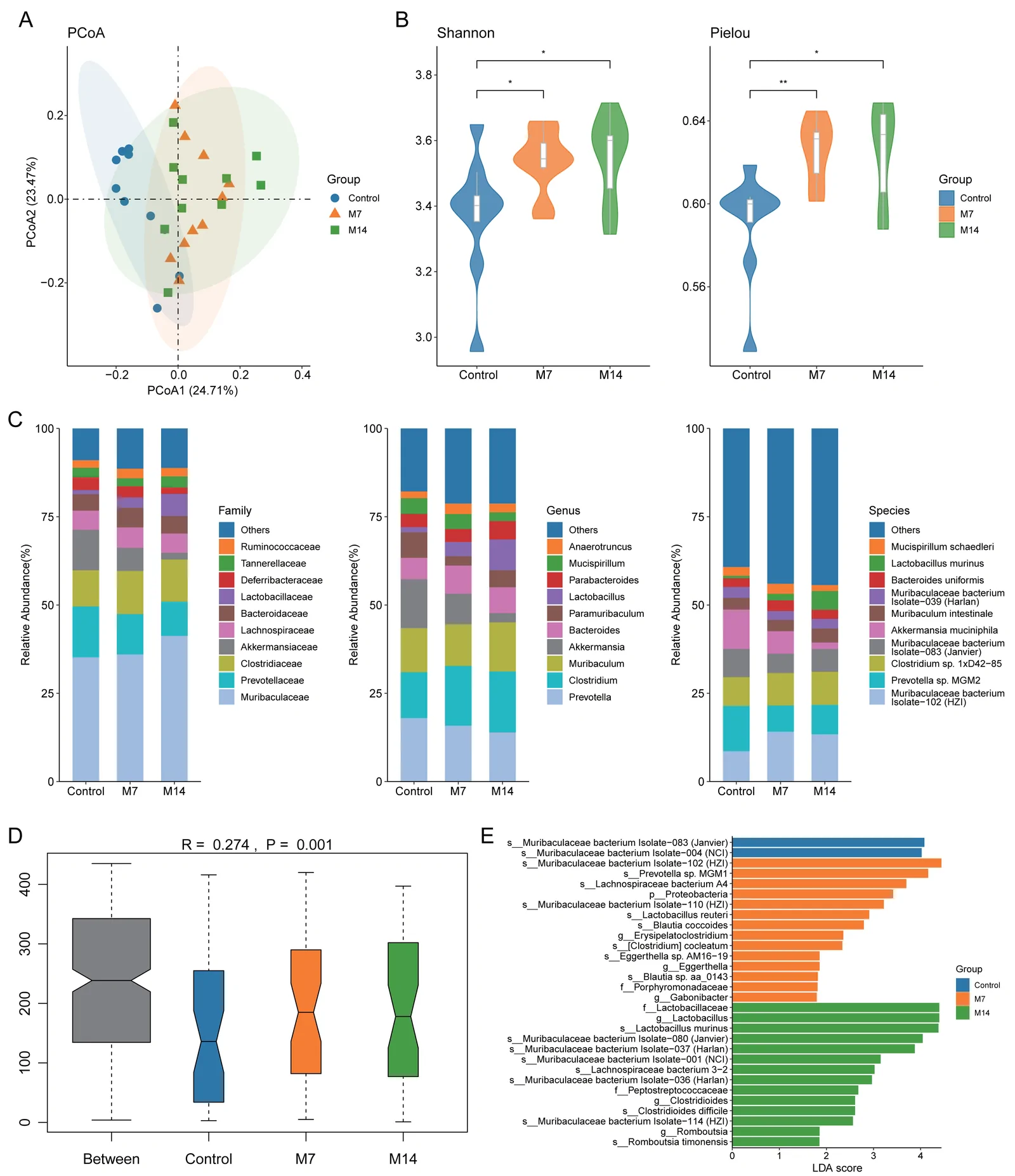
Compositional differences in gut microbiota of the mice with pulmonary fibrosis. (A) Principal coordinates analysis (PCoA) of gut microbiota composition across experimental groups. Beta diversity (Bray–Curtis distance) visualization showing microbial community clustering among Control (*n* = 10, blue), M7 (*n* = 10, orange), and M14 (*n* = 10, green) groups. Each point represents one individual mouse. Ellipses denote 95% confidence intervals for each group. (B) Alpha-diversity analysis of the gut microbiota among the Control, M7, and M14 groups at species level. Each point represents an individual animal (*n* = 10 per group); horizontal bars indicate mean; error bars represent standard error of the mean (SEM). Statistical analysis: Kruskal–Wallis test with Dunn’s post hoc correction. *p < 0.05 *versus* Control. (C) Relative abundance of dominant microbial taxa at Family, Genus, and Species levels. Stacked bar charts showing mean relative abundance (%) for each group. (D) The ANOSIM analysis quantifying inter-group *versus* intra-group differences in microbiota composition. *R*-statistic = 0.274, *p* = 0.001 (permutations = 999), indicating significant clustering by treatment group. (E) LEfSe analysis identifying significantly differential taxa among groups.

We performed metagenomic sequencing on fecal samples from distinct cohorts of mice: 10 Control (day 0), 10 M7 (day 7) and 10 M14 (day 14). The gut microbiota encompassed 79 families, 189 genera, and 554 species across samples. PCoA of Bray–Curtis distances indicated grouping differences among the three cohorts (Fig. 2A). Alpha diversity indices (Shannon, Pielou) differed across groups, with diversity measures increasing from baseline to day 14 (*P* < 0.05, Fig. 2B). Because separate cohorts were sampled at each time point and time-matched saline controls at days 7 and 14 were not included, these temporal trends should be interpreted cautiously: some shifts may reflect treatment-associated effects but could also reflect temporal drift, cage effects, or age-related changes.

At the family level, the predominant taxa included *Muribaculaceae*, *Prevotellaceae*, *Akkermansiaceae*, and *Clostridiaceae*, accounting for 64% to 71%. Notably, *Prevotellaceae* and *Akkermansiaceae* levels were significantly lower in the M7 and M14 groups than in the control group, while *Muribaculaceae* and *Clostridiaceae* levels were higher. At the genus level, the most abundant genera were *Prevotella*, *Akkermansia*, *Clostridium*, and *Muribaculum*, constituting 47% to 57%. Again, levels of *Prevotella* and *Akkermansia* were significantly lower in the M7 and M14 groups compared to the control group, while *Clostridium* was significantly higher. At the species level, the predominant species included *Prevotella sp. MGM2*, *Akkermansia muciniphila*, *Muribaculaceae bacterium Isolate-102 (HZI)*, and *Clostridium sp. 1xD42-85*, which accounted for 32% to 40%. Here too, *Prevotella sp. MGM2* and *Akkermansia muciniphila* levels were significantly lower in the M7 and M14 groups compared to the control group, while *Muribaculaceae bacterium Isolate-102 (HZI)* was significantly higher (Fig. 2C).

The analysis of similarities (ANOSIM) analysis further confirmed that inter-group differences were significantly greater than intra-group differences, indicating a profound effect of pulmonary fibrosis on gut microbiota composition (*R* = 0.274, *P* = 0.001, Fig. 2D), underscoring a profound impact of pulmonary fibrosis on gut microbiota composition. Together, these findings indicate that pulmonary fibrosis is associated with a restructuring of the gut microbiome characterized by loss of beneficial symbionts (Akkermansia and Prevotella) and expansion of other taxa, leading to increased overall microbial diversity that correlates with disease progression.

Subsequently, we conducted Linear Discriminant Analysis (LDA) Effect Size (LEfSe) analysis to identify significantly different bacteria between the control and model groups (M7 and M14) (Fig. 2E). In the M7 group, significant changes were observed in the abundances of *Muribaculaceae bacterium Isolate-102 (HZI)*, *Prevotella sp. MGM1*, and *Lachnospiraceae bacterium A4* (LDA >3.5). In the M14 group, *Lactobacillus* demonstrated significant changes at the genus level (LDA >4.0), while at the species level, the abundances of *Muribaculaceae bacterium Isolate-080 (Janvier)* and *Muribaculaceae bacterium Isolate-037 (Harlan)* were significantly altered (LDA >3.5).

### Significant differences in the functional potential of gut microbiota in mice with pulmonary fibrosis

**Figure 3:**
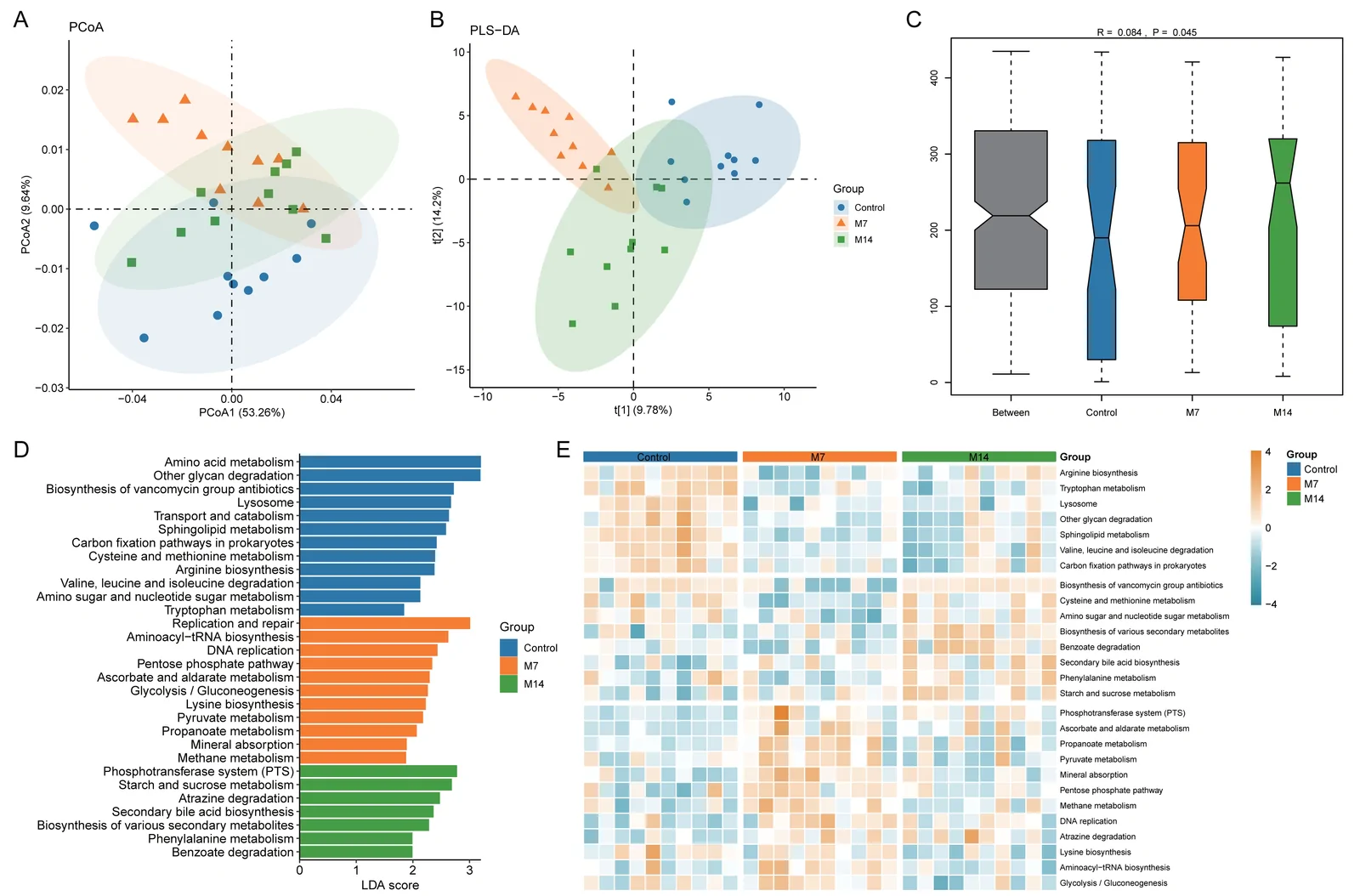
Predicted metabolic pathway alterations during bleomycin-induced fibrosis progression. (A) Principal coordinates analysis (PCoA) of predicted KEGG metabolic pathway representation across groups. Each point represents one animal (*n* = 10 per group); ellipses denote 95% confidence intervals. Shows spatial distribution of metabolic pathways with distinct separation among Control, M7, and M14 groups. (B) Partial Least Squares Discrimination Analysis (PLS-DA) of predicted KEGG metabolic pathway composition showing predictive modeling of functional gene category separation among groups (*n* = 10 per group). (C) The ANOSIM analysis quantifying inter-group *versus* intra-group differences in predicted metabolic pathway representation. *R*-statistic = 0.084, *p* = 0.045 (permutations = 999), indicating statistically significant differences in metabolic pathway composition among groups. (D) LEfSe analysis identifying significantly enriched predicted metabolic pathways by group. (E) Heatmap clustering analysis of differential KEGG predicted metabolic pathways. Orange indicates higher pathway representation; blue indicates lower representation.

To characterize predicted functional gene content, we performed KEGG and CAZy annotation and compared pathway representation across groups. PCoA and Partial Least Squares Discrimination Analysis (PLS-DA) based on predicted pathway profiles revealed group separation (Figs. 3A and 3B), and ANOSIM indicated greater inter-group than intra-group differences (*R* = 0.084, *P* = 0.045, Fig. 3C). LEfSe identified predicted pathway enrichments that varied by stage: Control samples were enriched for pathways related to amino acid metabolism, glycan biosynthesis and lipid metabolism; the M7 cohort showed altered representation of replication/repair and carbohydrate metabolism-related genes; and the M14 cohort exhibited increased representation of genes annotated to the phosphotransferase system (PTS), starch and sucrose metabolism, atrazine degradation, and secondary bile acid biosynthesis (LDA thresholds as indicated in the figure).

Subsequently, we performed a clustering analysis of these differential predicted pathways (Fig. 3E). The results revealed that pathways such as other glycan degradation, sphingolipid metabolism, valine, leucine, and isoleucine degradation, and carbon fixation pathways in prokaryotes maintained high activity levels in the control group, whereas their activity significantly decreased in the pulmonary fibrosis group. In the early stage of pulmonary fibrosis (M7), the activities of certain pathways—including the biosynthesis of vancomycin group antibiotics, cysteine and methionine metabolism, amino sugar, and nucleotide sugar metabolism—were reduced, but they returned to normal levels in the M14 stage. Conversely, pathways such as the PTS-ascorbate and aldarate metabolism, propanoate metabolism, pyruvate metabolism, and the pentose phosphate pathway exhibited transient increases in activity during the M7 stage, followed by a decrease to normal levels in the M14 stage. Again, these results reflect predicted genomic potential rather than measured metabolite fluxes.

### Changes in gut microbiota affect functional pathways

**Figure 4:**
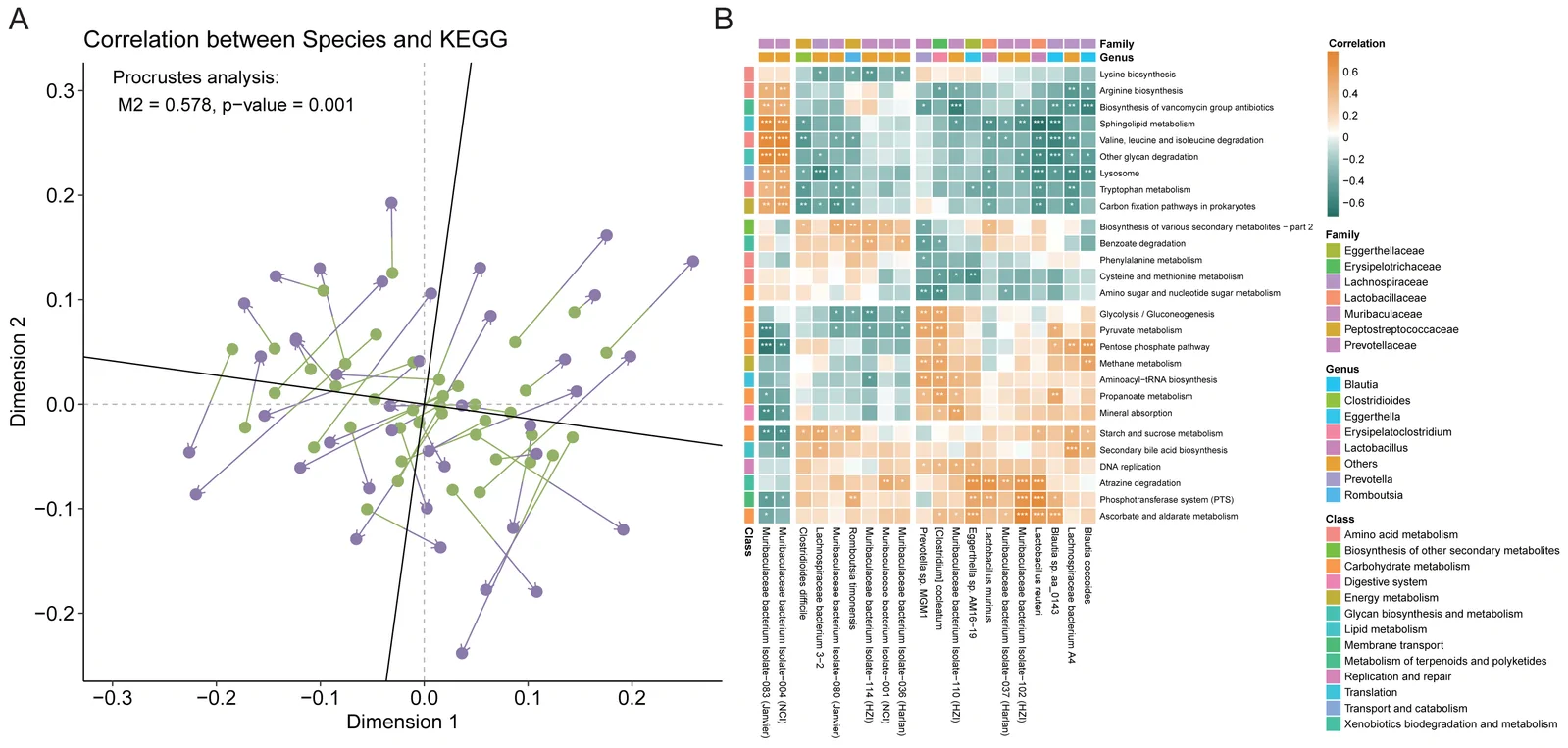
Association between gut microbiota composition and predicted metabolic pathway representation. (A) Procrustes analysis quantifying correspondence between gut microbiota composition and predicted KEGG metabolic pathway representation. Squared residual sum of discrepancies (*M*^2^) = 0.578. Permutation test (999 iterations) yielded *p* = 0.001, indicating statistically significant association between microbial community structure and predicted metabolic pathway representation. Lower *M*^2^ values indicate stronger correspondence. (B) Heatmap of Spearman rank-order correlations between differential microbiota and predicted KEGG pathways. Orange: positive correlation (*r* > 0); blue: negative correlation (*r* < 0).

To examine relationships between taxonomic composition and predicted functional potential, we performed Procrustes analysis linking microbial community structure to KEGG-based pathway profiles. The Procrustes M2 statistic was 0.578 (permutation *P* = 0.001), indicating a significant association between taxonomic variation and predicted pathway representation (Fig. 4A). Spearman correlation analysisbetween differentially abundant taxa and predicted pathways indicated that many taxa co-varied with specific predicted pathways: for example, predicted amino acid metabolism pathways tended to correlate negatively with several expanded taxa, while predicted starch/sucrose metabolism, secondary bile acid-related genes, atrazine degradation and PTS representation correlated positively with other taxa (Fig. 4B). These correlations identify candidate taxon–pathway links for experimental validation.

## Discussion

This study systematically explored the impact of pulmonary fibrosis on the gut microbiota and its downstream metabolic consequences in a murine model. In this study, we performed a stage-resolved, cross-sectional shotgun metagenomic analysis to characterize gut microbial taxonomic composition and predicted functional potential across early (day 7) and established (day 14) fibrosis stages in a bleomycin mouse model.

Through metagenomic sequencing, this study analyzed the changes in the composition of the intestinal microbiota in mice during the progression of pulmonary fibrosis. Compared to the normal group, the gut microbiota of mice in the M7 and M14 groups exhibited significant compositional differences in certain key families and genera (such as *Prevotellaceae*, *Akkermansiaceae*, *Muribaculaceae*, *Clostridiaceae*). Specifically, the relative abundances of *Prevotellaceae* and *Akkermansiaceae* in the M7 and M14 groups were significantly lower than in the normal group, while the relative abundances of *Muribaculaceae* and *Clostridiaceae* were significantly higher. These changes suggest that the progression of pulmonary fibrosis may be involved in alterations in specific members of the intestinal microbiota, thereby affecting the host’s metabolic state.

Moreover, the changes in the relative abundances of major genera in the gut microbiota (such as *Prevotella*, *Akkermansia*, *Muribaculum*, *Clostridium*) in the M7 and M14 groups were also closely related to the temporal shifts in these metabolic pathways. These alterations in the gut microbiota not only reflect the progression of pulmonary fibrosis but may also further promote or inhibit the pathological process of pulmonary fibrosis by modulating the host’s metabolic functions (Gong, Song & Su, 2021; Guo & Yang, 2024). A normal intestinal flora can maintain the integrity of the intestinal mucosa and prevent harmful substances from entering the body. When the flora is disordered, the intestinal barrier function is damaged, which may translocate inflammatory factors and other substances to enter the blood circulation, thereby acting on the lungs (Bowerman et al., 2020; Zhang et al., 2020). Through KEGG/CAZy-based functional annotation analysis, we identified predicted alterations in microbial metabolic pathway representation. Compared to the normal group, the metabolic pathways in the M7 and M14 groups showed significant differences in certain key metabolic pathways. For instance, in the M7 group, the functional potential of metabolic pathways related to replication and repair, as well as carbohydrate metabolism, were significantly reduced, which may align with the pathological characteristics of pulmonary fibrosis (Li et al., 2024; Liu et al., 2024). In contrast, in the M14 group, the functional potential of certain metabolic pathways (such as the PTS, starch and sucrose metabolism, atrazine degradation, and bile acid biosynthesis) were significantly increased, potentially reflecting a further deterioration of the pathological state of PF. The altered gut microbiota may modulate PF-related signaling pathways, potentially influencing the fibrotic process.

These changes in metabolic pathways may be closely related to the pathological features of PF, including enhanced inflammatory responses and accelerated fibrosis processes (Fan & Pedersen, 2021; Rajesh, Atallah & Barnthaler, 2023). Additionally, the dynamic changes in these metabolic pathways were also closely associated with the microbial community structure. Therefore, the results of this study support the notion that the gut microbiota may be involved in the progression of PF by modulating metabolic pathways.

Further analysis indicated that the alterations over time in certain metabolic pathways (such as amino acid metabolism, carbohydrate metabolism, and lipid metabolism) were closely related to specific members of the gut microbiota (Chen, Zhou & Wang, 2021; Fan & Pedersen, 2021; Gomaa, 2020). For example, in the normal group, the activities of key metabolic pathways like amino acid metabolism, carbohydrate metabolism, and lipid metabolism were relatively high, while in the M7 and M14 groups, the activities of these pathways changed as the progression of PF advanced. Additionally, some metabolic pathways exhibited phase-specific trends (*e.g.*, a decrease in the activity of certain metabolic pathways in the M7 group, while an increase was observed in the M14 group). Functional predictions based on metagenomic annotation remain hypothetical without metabolomic validation, necessitating confirmation through metabolomic analysis. As such, all pathway alterations presented here should be interpreted as predicted metabolic capacity rather than confirmed metabolic activity.

Collectively, our data are consistent with an association between bleomycin-associated PF and compositional changes in the gut microbiota, accompanied by alterations in predicted microbial functional potential. These microbial–predicted functional changes may contribute to host metabolic alterations and represent candidate biomarkers or targets, but causal linkage remains to be established. These microbial–metabolic interactions may not only serve as biomarkers of disease progression but also represent actionable targets (Rajesh, Atallah & Barnthaler, 2023; Sun et al., 2024b). Fecal microbiota transplantation can alter the composition of the intestinal microbiota in patients with PF, increase beneficial flora, reduce harmful flora, improve intestinal barrier function, and may perturb PF-related signaling pathways to inhibit the fibrotic process.

Nevertheless, several limitations warrant consideration. First, the experimental design was cross-sectional: separate cohorts were sampled at baseline, day 7 and day 14 rather than repeatedly sampling the same individuals. Second, saline-treated control animals were sampled only at baseline, and time-matched vehicle controls at days 7 and 14 are absent. Therefore, some observed differences between baseline and later time points may reflect temporal drift, cage or environmental effects, age-related changes, or handling differences rather than being solely attributable to bleomycin-induced fibrosis. Third, functional analyses were based on KEGG and CAZy annotation of gene content and thus reflect predicted metabolic potential rather than measured metabolite levels; we did not perform fecal, serum, or lung metabolomics in this study. Fourth, although histology and immunohistochemistry (TGF-*β*1, *α*-SMA) support fibrogenesis, more quantitative fibrosis metrics (*e.g.*, Ashcroft scoring, hydroxyproline/collagen quantification, lung qPCR/protein assays) were not available and would strengthen correlations between fibrosis burden and microbiota changes. Finally, causality was not tested here: experiments such as microbiota depletion (antibiotic treatment or germ-free models), fecal microbiota transplantation, or targeted metabolite supplementation are needed to establish whether microbial alterations contribute to PF pathogenesis or are secondary consequences of disease. We explicitly note these limitations and recommend that future studies include time-matched vehicle controls, longitudinal within-subject sampling, direct metabolomic measurements, and causality experiments.

## Conclusion

In summary, our stage-resolved cross-sectional analysis identifies taxa and predicted metabolic pathways that associate with experimentally induced pulmonary fibrosis, offering hypotheses and candidate targets for future mechanistic and translational studies.

##  Supplemental Information

10.7717/peerj.21693/supp-1 Supplemental Information 1 Supplemental File

10.7717/peerj.21693/supp-2 Supplemental Information 2 ARRIVE Checklist

## Additional Information and Declarations

### Data Availability

The following information was supplied regarding data availability: The raw data is available in the Supplemental Files. The code is available at Zenodo: - Yang, X. (2026). Gut Microbiota Dynamics and Metabolic Pathways Associated with Bleomycin-Induced Pulmonary Fibrosis Progression. Zenodo. https://doi.org/10.5281/zenodo.20910771
The datasets are available at the China National GeneBank Sequence Archive (CNSA): CNP0007957.
